# Hypovitaminosis D in HIV-infected patients in Lisbon: a link with antiretroviral treatment

**DOI:** 10.7448/IAS.17.4.19826

**Published:** 2014-11-02

**Authors:** Márcia Boura, Ana Filipa Sutre, Robert Badura, Alexandra Zagalo, Cláudia Afonso, Luís Caldeira, Emília Valadas

**Affiliations:** 1 Clínica Universitária de Doenças Infecciosas, Faculty of Medicine, University of Lisbon, Lisbon, Portugal; 2 Clínica Universitária de Doenças Infecciosas, Faculty of Medicine, Santa Maria University Hospital, University of Lisbon, Lisbon, Portugal

## Abstract

**Introduction:**

Recent data indicates that low vitamin D (25(OH)D) levels can lead to a worst prognosis in HIV-infected individuals, even in those on successful antiretroviral therapy (ART) [1]. Portugal is the European country that has the largest average sun exposure time but prevalence of hypovitaminosis D is mostly unknown. Our aim was to determine the prevalence of hypovitaminosis D in HIV patients in Lisbon and the possible association with ART.

**Methods:**

From 2012 to January 2014, plasma samples from 518 HIV-infected patients were collected to 25(OH)D levels determination. Data on demographic features (age, ethnicity, country of origin) and clinical/laboratory parameters were collected from clinical files (HIV subtype, CD4+ cell count, CD4+ nadir, viral load (VL), HBV/HCV co-infection and ART). 25(OH)D status was defined as: deficiency <20 ng/mL, insufficiency 20–30 ng/mL, optimal >30 ng/mL.

**Results:**

Median age was 46 years old (±11); 62.0% (321/518) were male; 81.3% (421/518) were Caucasian and 78.6% (407/518) were Portuguese. Most patients (96.1%; 498/518), were HIV-1 infected, 22.9% (114/498) and 4.0% (20/498) of them were HCV and/or HBV co-infected, respectively. Mean CD4+ cell count was 648 cells/µL (±333) and nadir was 219 cells/µL (±179). On treated patients VL was <40 HIV RNA/mL in 86.7% (417/481). The median levels of 25(OH)D was 20.0 ng/mL (range 4.1–99.7) and we found differences between values observed during Winter (median 16.7 ng/mL) and Summer (median 24.9 ng/mL) (p<0.0001). Low 25(OH)D levels were not correlated to ethnicity (p=0.066). 25(OH)D level was <30 ng/mL in 80.1% (415/518) of the patients, from which 30.9% (160/518) and 49.2% (255/518) had insufficiency and deficiency levels, respectively. Most (92.9%; 481/518) were on ART: regimens containing PI (47.5%), NNRTI (40.3%; 41.3% on NVP and 58.7% on EFV), II (1.2%), PI+NNRTI (3.9%). Comparing the 25(OH)D level along the different ART regimens (PI vs NVP; PI vs EFV; PI vs no ART) there were differences between PI and EFV (p=0.044).

**Conclusions:**

In this study, 80.1% of the HIV-infected patients had hypovitaminosis D and ART regimens with EFV were more often associated with low 25(OH)D levels. Understanding the impact of the different antiretroviral drugs on 25(OH)D status could help to decide in clinical practice whether 25(OH)D supplementation or drug switch are the best options for each patient.
